# Circulating DNA as biomarker in breast cancer

**DOI:** 10.1186/s13058-015-0645-5

**Published:** 2015-10-09

**Authors:** Heidi Schwarzenbach, Klaus Pantel

**Affiliations:** Department of Tumor Biology, University Medical Center Hamburg-Eppendorf, Martinistraβe 52, 20246 Hamburg, Germany

## Abstract

As the release of tumor-associated DNA into blood circulation is a common event in patients with cancer, screening of plasma or serum DNA may provide information on genetic and epigenetic profiles associated with breast cancer development, progression, and response to therapy. Quantitative testing of circulating DNA can reflect tumor burden, and molecular characterization of circulating DNA can reveal important tumor characteristics relevant to the choice of targeted therapies in individual patients. Contrary to circulating DNA from blood that presents molecular changes in tumor DNA in real time, tissue biopsies can deliver only a spatially and temporally limited snapshot of the heterogeneous tumor. Analyses of circulating DNA might provide prognostic and predictive information and therefore advance personalized medicine. However, standardization of different technical platforms as well as the control of pre-analytical and analytical factors is mandatory before its introduction into clinical practice. In the present review, we discussed technical aspects and clinical relevance of the analyses of circulating plasma/serum DNA in patients with breast cancer.

## Introduction

The presence of circulating, cell-free nucleic acids in the bloodstream was first described by Mandel and Métais in 1948 [1]. Thirty years later, Leon et al. showed that patients with breast cancer (BC) display increased serum levels of cell-free DNA (cfDNA) in their blood circulation; they detected a wide DNA range, from 0 to 2000 ng/ml, by radioimmunoassay [2]. In 1999, Silva et al. detected genetically altered and methylated cfDNA in the plasma of patients with BC [3–5]. These observations led to intensive quantitative and qualitative investigations of cfDNA in patients with BC.

The release of cfDNA into the bloodstream occurs by different sources, including the primary tumor, tumor cells that circulate in peripheral blood, metastatic deposits present at distant sites, and normal cell types, such as hematopoietic and stromal cells. Thus, both tumor and normal cfDNA circulate in the bloodstream of patients with cancer. Its rapidly increased accumulation in blood during tumor development is caused mainly by an excessive DNA release by apoptotic and necrotic cells. In addition, active secretion within exosomes has been demonstrated [6], but it is still discussed whether this is a relevant or rather minor source of cfDNA. The reduced clearance of cfDNA caused by impaired organ function during systemic inflammation may also contribute to cfDNA elevation in the blood of patients with cancer [7]. Because of the reduced cell turnover and more efficient removal of defect cells from the circulation by phagocytes, the concentrations of cfDNA are low in healthy individuals. Usually, cfDNA is removed from blood by liver and kidney, and its half-life is 10 to 15 min [8, 9]. The size of cfDNA may indicate its source. Apoptotic cells produce DNA fragments of 180–200 base pairs (bp) or multiples of this unit, whereas necrotic cells release higher molecular-weight DNA fragments of over 10,000 bp in size [10]. In patients with BC, cfDNA has also been analyzed in sources other than plasma and serum, such as urine [11]. Additionally, cfDNA species, such as cell-free mitochondrial DNA, are also under evaluation for clinical relevance [12].

In blood, cfDNA circulates predominantly as nucleosomes, which are nuclear complexes of histones and DNA [13]. They are frequently nonspecifically elevated in cancer but may be most valuable for monitoring cytotoxic cancer therapy, particularly for the early estimation of therapy efficacy. Combinations with other oncological or apoptotic biomarkers have been suggested to improve the sensitivity of detecting nonresponse to cytotoxic chemotherapy. Moreover, during neoadjuvant chemotherapy, BC patients with no change of disease had significantly higher pretherapeutic levels of circulating nucleosomes than patients in remission, indicating that apoptotic biomarkers bear valuable information for diagnosis and therapy response prediction [14].

BC is associated with different genetic and epigenetic events, such as DNA strand integrity, gene amplifications, gene mutations, DNA methylation, and microsatellite abnormalities. These alterations detected in the primary tumor may also be found in plasma/serum cfDNA of patients with BC (Table 1). In the present overview, we will concentrate mainly on recent progress in blood-based cfDNA analyses in BC.

**Table 1:** Selected studies on circulating DNA in plasma and serum of patients with breast cancer

Number of patients	Target of analysis	Methods	Clinical relevance	Reference
52 M0, 26 benign	Mitochondrial DNA quantification	qPCR	Detection of cancer	[12]
			Deregulated DNA levels	
			Diagnosis	
51 M0, 28 M1, 13 benign	Nucleosome quantification	ELISA	Monitoring for therapy response	[14]
			Deregulated nucleosome levels	
31 M0, 32 M1, 20 benign	DNA, nucleosome quantification	PicoGreen, ELISA	Detection of cancer progression	[13]
			Elevated nucleosome/DNA levels	
			Diagnosis	
64 M0	DNA quantification	qPCR	Monitoring for MRD	[22]
			Inverse relationship with DTC status	
100 Stage I–IV	DNA quantification	Fluorometer	Detection of cancer	[19]
			Elevated DNA levels	
			Diagnosis	
61 M0, 33 benign	DNA quantification	qPCR	Screening for early detection and follow-up	[20]
			Deregulated DNA levels	
			Diagnosis	
83 M0	DNA integrity	qPCR	Detection of cancer progression	[24]
			Diagnosis	
65 M0, 47 M1, 12 benign	DNA integrity	qPCR	Detection of cancer	[25]
			Diagnosis	
82 M0, 201 M1	DNA integrity	qPCR	Detection of cancer	[26]
			Correlation with progression-free and overall survival	
			Diagnosis and prognosis	
65 M0	DNA integrity	qPCR	Monitoring for therapy response	[27]
25 M0	Mutations	PCR-SSCP and direct sequencing	Detection of cancer	[5]
			Diagnosis	
313 M0	Mutations	Digital PCR	Correlation with recurrence-free and overall survival	[33]
			Prognosis	
17 M1	Mutations	Next-generation sequencing	Detection of metastasis	[34]
			Diagnosis	
33 M0	Mutations	Digital PCR	Detection of cancer	[35]
			Diagnosis	
30 M1	Mutations	Targeted sequencing	Detection of metastasis	[36]
			Monitoring for therapy response	
2 M0	Mutations	Whole exome sequencing	Detection of acquired drug resistance in advanced cancer	[37]
65 M0	SNP/CNV	Array	Detection of cancer during routine follow-up	[31]
			Correlation with MRD	
			Diagnosis	
58 M1	Copy number	Whole-genome sequencing	Dynamic variation of DNA range in metastasis	[38]
65 M0, 58 M1	Copy number	Digital PCR	Screening for the acquisition of HER2 amplification in metastasis	[29]
102 M0, 32 benign	LOH	PCR	Correlation with overall and disease-free survival	[21]
			Diagnosis and prognosis	
62 M0	LOH, Methylation, Mutations	Microsatellite, PCR-SSCP, MSP	Detection of tumor progression	[3]
			Diagnosis	
35 M0	Methylation	MSP	Detection of cancer	[4]
			Diagnosis	
428 M0	Methylation	MethyLight PCR	Correlation with overall and disease-free survival	[39]
			Therapy-independent prognosis	
101 M0, 58 M1	Methylation	OS-MSP	Detection of metastasis	[40]
			Diagnosis	
336 M0	Methylation	OS-MSP	Correlation with overall survival	[41]
			Prognosis	
80 M1	Methylation	MSP	Correlation with CTC status	[42]
148 M0	Methylation	MethyLight PCR	Monitoring for therapy response	[43]
52 M0	Methylation	MSP	Monitoring for therapy response	[44]
110 M0	Methylation	MSP	Detection of estrogen receptor-negative status	[45]
120 M0, 100 benign	Methylation	MSP	Detection of cancer	[46]
			Diagnosis	
155 M0	Methylation	MSP	Detection of metastasis	[47]
			Correlation with overall and disease-free survival	
			Diagnosis and prognosis	
79 M0	Methylation	MSP	Correlation with CTC status	[48]
			Diagnosis	
100 M0	Methylation	MSP	Correlation with protein expression	[50]
			Diagnosis	
203 M0	Methylation	MSP	Association with DNA repair capacity	[51]
			Diagnosis	
304 M0 234 benign	Methylation	Pyrosequencing	Modest difference in methylation patterned	[52]

This table represents a selection of cell-free DNA analyses in plasma or serum of patients with breast cancer and is not meant to be comprehensive. It is based on our own view of studies that offer substantial clinical insight

*CNV* copy number variation, *CTC* circulating tumor cell, *DTC* disseminated tumor cell, *ELISA* enzyme-linked immunosorbent assay, *HER2* human epidermal growth factor receptor 2, *LOH* loss of heterozygosity, *M0* patients with primary breast cancer, *M1* patients with metastatic breast cancer, *MRD* minimal residual disease, *MSP* methylation-specific polymerase chain reaction, *OS-MSP* one-step methylation-specific polymerase chain reaction, *PCR-SSCP* polymerase chain reaction-single strand conformation polymorphism, *qPCR* quantitative real-time polymerase chain reaction, *SNP* single-nucleotide polymorphism

## Techniques

### Quantification

cfDNA has been quantified by fluorescence-based methods, such as PicoGreen staining and ultraviolet spectrometry, or by the more sensitive quantitative polymerase chain reaction (PCR; SYBR Green or TaqMan) of repetitive elements or housekeeping genes [15]. Circulating nucleosomes, which are the primary repeating unit of DNA organization in chromatin, have usually been quantified by enzyme-linked immunosorbent assays [13].

### Genetic analyses

Microsatellite instability has routinely been assessed by using microsatellite-based fluorescence PCR. Mutations and copy number in cfDNA have been probed by using allele-specific PCR assays. However, for detection of rare mutations, more sensitive techniques, such as BEAMing (beads, emulsion, amplification and magnetics) or PAP (pyrophosphorolysis-activated polymerization), as well as digital genomic technologies adjusted with deep or next-generation sequencing DNA, are increasingly applied. A detailed overview of these technologies has been published elsewhere [16].

### Epigenetic analyses

Aberrantly methylated cytosines within CpG dinucleotides can be detected by sodium bisulphite treatment of extracted cfDNA, which converts unmethylated cytosines to uracil. Techniques such as methylation-specific PCR, methyl-BEAMing, and quantum dot-based fluorescence resonance energy transfer (QD-FRET) have recently been adapted for the detection of methylated cfDNA (reviewed in [17]).

Table 2 summarizes the advantages and limitations of the currently used technologies to analyze cfDNA. However, these technical platforms differ in sensitivity and specificity, and their technical limitations to detect low levels of tumor cfDNA in an excess of wild-type DNA, as well as their sequence-specific bias, influence the results and consequently their comparability.

**Table 2:** Commonly used technologies to analyze cell-free DNA

Technique	Advantages	Limitations
Quantitative PCR	High sensitivity and specificity	Quantification of only annotated sequences
	High dynamic range	
BEAMing, PAP, COBRA, etc.	Higher sensitivity and specificity than quantitative PCR	Analyses of only predetermined sequences
	Detection of at least 0.01 % altered alleles	
Microarray	High throughput	Not suitable for accurate quantification
	Relatively low cost	Low dynamic range
	Detection of only annotated DNA	High signal-to-noise ratio
	Cross-hybridization between similar sequences	
Next-generation sequencing	High sensitivity and specificity	High cost
	Detection of novel and rare alterations	Need for special equipment and bioinformatics
	Ability to distinguish similar sequences	Relatively high amounts of starting material
		Sequence-specific bias

*PCR* polymerase chain reaction, *BEAMing* beads, emulsion, amplification and magnetics, *PAP* pyrophosphorolysis-activated polymerization, *COBRA* combined bisulfite restriction analysis

## cfDNA quantification and integrity

The possibility of using plasma/serum cfDNA concentrations as an indicator of BC has been investigated by numerous studies [18]. Using fluorometry, Tangvarasittichai et al. showed the continuous increase in plasma cfDNA during tumor progression and its decrease after surgery. The median concentrations of plasma cfDNA were 0.5, 235, 422, 1280, and 0.5 ng/ml in BC patients classified by tumor stage I, II, III, and IV and surgical patients, respectively [19]. Huang et al. found that the median plasma DNA concentration (65 ng/ml) was significantly higher in patients with BC than in patients with benign breast diseases (22 ng/ml) or healthy women (13 ng/ml) [20]. A similar cfDNA elevation was documented by Kohler et al. They found that the cfDNA levels were significantly higher in patients with BC than in patients with benign diseases and healthy women, whereas the mitochondrial cfDNA levels were lower in both tumor cohorts [12]. In contrast, we found that although the serum cfDNA levels in patients with BC were significantly higher than in healthy women and were associated with a poor overall and disease-free survival, they could not discriminate malignant from benign breast lesions [21]. These discrepancies in the quantification of cfDNA may be explained by the use of plasma or serum and the different technical platforms, and emphasize that in the future a standardization of cfDNA measurements is essential.

Payne et al. [22] dealt with BC dormancy, the time between removal of the primary tumor and subsequent relapse of patients who have been clinically disease-free [23]. The authors quantified two overlapping cfDNA (96- and 291-bp) amplicons in the plasma of patients with BC. Increasing cfDNA concentrations correlated with estrogen receptor (ER), HER2, triple-negative tumors, and high tumor grade. Besides, an inverse relationship between mRNA of the epithelial marker cytokeratin 19 in bone marrow and the 291-bp amplicon in cfDNA was observed, suggesting that an inverse relationship between cell viability of disseminated tumor cells in bone marrow and cell death in plasma, respectively, occurs during the dormancy phase of BC [22].

Quantification of cfDNA has been extended to measurements of the integrity of cfDNA. Umetani et al. quantified the serum DNA integrity of short and long cfDNA fragments of noncoding *ALU* repeat sequences, which are interspersed on chromosomes throughout the genome. Mean serum DNA integrity was significantly higher in patients with BC than in healthy women and was associated with lymphovascular invasion, lymph node metastasis, and tumor size. The receiver operating curve for discriminating lymph node status had an area under the curve (AUC) of 0.81. These findings indicate that an *ALU* DNA integrity assay is sensitive to detect early-stage metastasis to regional tumor-draining lymph nodes [24]. Stotzer et al. demonstrated that plasma cfDNA is rather valuable for the detection of locally confined BC but that the established tumor markers are most informative in metastatic BC [25]. Discrimination of locally confined BC from healthy controls was achieved by *ALU115* (AUC of 95.4) and *ALU247* (AUC of 95.5) and of metastatic BC from both benign controls and locally confined BC by CA15-3 (AUC of 83.2) and CEA (AUC of 79.1). Moreover, Madhavan et al. determined plasma cfDNA concentrations and cfDNA integrity of *ALU* and *LINE1* repetitive DNA elements [26]. A hierarchical decrease in cfDNA integrity and increase in cfDNA concentrations from healthy controls over primary BC and further to patients with metastatic BC were observed. Combination of cfDNA integrity and concentrations could differentiate controls from primary BC cases (AUC of 0.75), circulating tumor cell (CTC)-negative metastatic cases (AUC of 0.81), and CTC-positive metastatic BC (AUC of 0.93) as well as CTC-negative from CTC-positive metastatic BC cases (AUC of 0.83). The cfDNA integrity was additionally associated with progression-free and overall survival of patients with metastatic BC and had a lower prediction error than the CTC status [26].

cfDNA measurements have also been carried out in BC patients undergoing chemotherapy. Patients with large or nonoperable BC often receive neoadjuvant chemotherapy to facilitate full resection of the tumor and enable conservation of breast. Lehner et al. quantified the levels of repetitive *ALU115* and *ALU247* elements in such patients with BC before surgery and found that the pretherapeutic HER2 status was positively correlated with therapy response [27]. From therapy cycle 1 to 6, the kinetics of *ALU115* showed decreases in patients with complete remission, whereas in patients with no change of disease, an increase was observed. Similar tendencies were obtained for *ALU247* fragments, whereas CEA and CA15-3 were not informative for therapy outcome.

The studies described above are not claimed to be comprehensive, but they provide insight into the clinical relevance of cfDNA levels and integrity in different populations of patients with BC. They demonstrate that their quantifications may serve as a diagnostic, prognostic, and predictive marker for BC. However, the increase in cfDNA levels is not cancer-specific, because their changes can also be observed in other cancer types. Moreover, their values can overlap between benign and malignant diseases. Therefore, these measurements may be rather useful in combination with other blood tumor biomarkers.

## Genetic analyses

Microsatellite instability, such as loss of heterozygosity (LOH), is frequently found in tumor tissues. To date, LOH has also been detected in plasma or serum cfDNA and its frequency correlated with diagnosis and prognosis in patients with BC (reviewed in [18]). Recently, however, the interest in analyzing LOH in cfDNA has decreased because, owing to the high background of normal cfDNA in blood, the LOH frequency was usually low. Owing to inflammation or tissue repair processes, leukocytes and stroma cells, as well as the abnormal proliferation of benign cells, contribute to the accumulation of normal wild-type DNA in blood. The proportion of wild-type DNA in the blood circulation of patients with BC is unknown and may fluctuate. Therefore, only a small fraction of cfDNA is tumor-derived (reviewed in [18]).

At present, in the scientific community, there is an increased focus on the analyses of cfDNA copy number and mutations using new technical platforms (reviewed in [28]). In this regard, digital PCR was adapted to determine the presence of *HER2* copy number in plasma cfDNA. Using this approach, Gevensleben et al. showed that the plasma cfDNA copy number ratio of *HER2* to a reference gene had an AUC of 0.92 and that 64 % of patients with HER2-amplified cancer and 94 % of patients with *HER2*-nonamplified cancer displayed positive and negative predictive values of 70 % and 92 %, respectively [29].

In disease relapse and formation of metastases, minimal residual disease (MRD), usually analyzed by CTCs, plays a major role [30]. To monitor MRD by copy number variations, Shaw et al. profiled 251 genomes using Affymetrix genome-wide human single-nucleotide polymorphism arrays [31]. In paired cfDNA and primary tumor samples, focal high-level DNA amplifications clustered in numerous chromosome arms, some of which harbored genes with oncogenic potential, including *USP17L2* (*DUB3*), *BRF1*, *MTA1*, and *JAG2*, were identified. Up to 12 years after diagnosis, these amplifications were still detectable in some of the follow-up plasma samples. These cfDNA array analyses could distinguish between preoperative BC patients and those who have had surgery and treatment.

In ER-positive BC, mutations of *PIK3CA* are the most frequent genomic alterations. They lead to activation of the phosphoinositide 3-kinase (PI3K) signaling pathway that plays a central role in cellular processes, such as cell survival, growth, division, and motility [32]. Applying a digital PCR assay, Oshiro et al. detected that serum samples from 23 % of *PIK3CA*-mutant patients were positive for this mutation [33]. No *PIK3CA*-mutant cfDNA was detected in the serum samples of *PIK3CA*-nonmutant BC patients and healthy women. The dichotomization of patients into *PIK3CA*-mutant subgroups with high and low cfDNA levels showed that the mutant cfDNA-high level subgroup exhibited significantly shorter recurrence-free and overall survival rates than the cfDNA-low level subgroup and nonmutant patient group, indicating that *PIK3CA* mutation status is a significant and independent prognostic factor for patients with BC [33].

However, the detection rate of mutations is higher if the new technique of next-generation sequencing (NGS) is applied. When plasma samples were analyzed by Rothe et al., 12 of 17 patients had more than one mutation in *p53*, *PIK3CA*, *PTEN*, *AKT1*, *IDH2*, or *SMAD4*. In 13 of 17 patients, plasma and tumor tissue samples provided concordant results, whereas the four other discordant cases provided complementary information [34]. Bettegowda et al. used whole-genome sequencing and detected tumor cfDNA in the plasma of more than 75 % of advanced BC and 50 % of localized BC patients [35]. The frequent presence of tumor cfDNA in BC patients without detectable CTCs suggests that these two circulating biomarkers are distinct entities.

Finally, using targeted or whole-genome sequencing, the laboratory of Caldas and Rosenfeld identified somatic genomic alterations in serially collected plasma from patients with metastatic BC [36]. Tumor-associated cfDNA was detected in 97 % of BC patients in whom somatic genomic alterations were identified, whereas the tumor marker CA15-3 and CTCs were detected in 78 % and 87 % of the patients, respectively. The tumor cfDNA levels showed a greater dynamic range, and greater correlation with changes in tumor burden, than did CA15-3 or CTCs detected by the CellSearch system. It should be mentioned that, despite the ground-breaking nature of this study, the dropout rate was considerable and the analyses were restricted to a small cohort of patients. Therefore, these results need further validation. The same group also massively parallel sequenced cancer exomes in serial plasma samples to track genomic evolution of metastatic BC in response to therapy. Quantification of allele fractions in plasma identified increased representation of mutant alleles in association with acquired drug resistance. These included an activating mutation in *PIK3CA* following treatment with paclitaxel, a truncating mutation in the ER co-activator *MED1* (mediator complex subunit 1) following treatment with tamoxifen and trastuzumab and following subsequent treatment with lapatinib, a splicing mutation in *GAS6*, the ligand for the tyrosine kinase receptor AXL, in the same patient [37].

To sum up, the inconsistent detection of mutations in cfDNA by the earlier studies could be improved by advanced genomic approaches that have higher sensitivity to identify rare mutations in matched cfDNA and tumor tissue samples. However, the study by Heidary et al. exemplified that cfDNA content may not always reflect the actual stage of disease progression [38]. Although all patients had progressive metastatic disease, plasma analyses demonstrated highly variable allele fractions of mutant fragments. The allele fractions of cfDNA were only 2–3 %, neither reflecting the tumor burden nor the dynamics of the disease, whereas the number of CTCs was exceedingly high, indicating that analysis of cfDNA and CTCs provides complementary information. The variable presence of mutated plasma cfDNA, together with high-resolution fragment sizing, was explained by differences in phagocytosis and DNA degradation mechanisms [38]. Moreover, progressing metastatic lesions consist mainly of viable cells and may have only a small fraction of apoptotic cells that are able to release DNA into the circulation.

## Epigenetic analyses

Aberrant DNA methylation is thought to be associated with an increased risk for neoplastic transformation. By the hypermethylation of CpG-rich regions in their promoter region, tumor suppressor genes become inactivated. Although such epigenetic alterations are not unique for BC, there are tumor suppressor genes that are frequently methylated and downregulated in BC. A phenotypic feature of BC biology is methylated *RASSF1A*. Its aberrant promoter hypermethylation may be common among high-risk women, leading to the repression of this scaffold protein that localizes signaling in cells. Gobel et al. investigated the methylation status of *RASSF1A* together with *PITX2* in the blood plasma and bone marrow plasma of patients with BC by using MethyLight, a quantitative methylation-specific PCR method [39]. Methylated *RASSF1A* and *PITX2* detected in blood plasma were significant indicators for poor overall survival and distant disease-free survival, whereas in bone marrow plasma only methylated *RASSF1A* had a prognostic value. Yamamoto et al. showed that the sensitivity of detection of methylation in *RASSF1A*, *GSTP1*, or *RARβ2* (or a combination of these genes) in the serum of patients with primary and metastatic BC was significantly higher than that involving CEA or CA15-3 [40]. When the assay was combined with these conventional tumor markers, the sensitivity increased to 78 %. Fujita et al. showed that 10 % of patients were positive for methylated serum cfDNA of these three genes and also had a significantly worse overall survival rate at 100 months in comparison with those with negative findings [41]. When combined with the levels of total serum DNA, these methylated cfDNA concentrations were even more effective for prediction of prognosis for patients with BC. Furthermore, Van der Auwera et al. demonstrated that detection of methylated *RASSF1A* (29 %), adenomatous polyposis coli (APC) (35 %), or *ER1* (20 %) correlated with the number of CTCs, enumerated by the CellSearch System, and thus indicates the CTC status in patients with metastatic BC [42].

Detection of methylated *RASSF1* in blood may also monitor response during chemotherapy. The methylation status of *RASSF1A* has also been investigated under chemotherapy. In this regard, Fiegl et al. demonstrated that *RASSF1A* methylation is an independent predictor of poor outcome for patients with BC [43]. The authors analyzed *RASSF1A* methylation in BC patients who received adjuvant tamoxifen. In their serum samples before therapy and 1 year after surgery, 19.6 % and 22.3 % of patients with BC harbored *RASSF1A* methylation, respectively. Disappearance of *RASSF1A* methylation in the serum throughout treatment with tamoxifen indicated response, whereas its persistence or new appearance meant resistance to adjuvant tamoxifen treatment. In the pilot study by Avraham et al., consecutive serum samples from 52 patients with locally advanced BC were analyzed for *RASSF1* methylation during neoadjuvant chemotherapy [44]. Methylated *RASSF1* was detected in the serum of 40 % of patients before therapy. In 8 % of patients who achieved complete pathological response, *RASSF1* methylation became undetectable in the serum early during therapy. In contrast, in 33 % of patients who had partial or minimal pathological response, *RASSF1* methylation persisted in the serum longer or throughout the treatment, respectively [44].

*ER* status is an important predictive marker, and its loss leads to the resistance to endocrine treatment in patients with BC. In this regard, Martinez-Galan et al. explored the relationship between ER expression in tumor tissue and methylation of the *ER* promoter region in plasma [45]. A significant association between methylated *ER* in cfDNA and ER-negative status was detected, indicating silencing by methylation of *ER* in BC. In the study by Hagrass et al., three different markers (*ER3*, *ER4*, and *ER5*) were used to analyze methylation status of the *ERα* promoter region in serum and tumor tissue samples [46]. The rates of methylation status of *ER3*, *ER4*, and *ER5* were 65 %, 26.7 %, and 61.7 % in tumor tissue, respectively, and 57.5 %, 21.7 %, and 55.8 % in serum, respectively, indicating the lower frequency of DNA methylation in serum. The rates of concordance between tumor and serum DNA methylation were 80 %, 72 %, and 92 % for *ER3*, *ER4*, and *ER5*, respectively, suggesting that methylated DNA is released mainly by the primary tumor.

The transcription factor Sox17 is involved in a variety of developmental processes and can act as an antagonist of the canonical Wnt/beta-catenin signaling pathway. Fu et al. examined the methylation status of *Sox17* in paired BC tissue and plasma samples [47]. The frequencies of Sox17 methylation were 72.9 % in BC tissues and 58.1 % in plasma cfDNA. Methylated *Sox17* was not found in normal breast tissues and plasma samples. The significant correlation of *Sox17* methylation between tumor tissues and paired plasma was associated with tumor node metastasis stage, lymph node metastasis, poor disease-free survival, and overall survival, indicating that Sox17 methylation may be an independent diagnostic and prognostic factor for BC. Chimonidou et al. found a relationship of this highly methylated gene with the presence of CTCs in BC patients after surgical removal of the primary tumor [48]. The *SOX17* promoter was methylated in 86.0 % of tumor tissues. In CTCs, *SOX17* was methylated in 34.5 % of patients with early BC, 45.8 % of patients with metastatic cancer, and one healthy woman, whereas in matched plasma cfDNA samples, *SOX17* was methylated in 34.5 %, 40.7 %, and one of the corresponding individuals, respectively. There was a significant correlation between *SOX17* methylation in cfDNA and CTCs in patients with early BC but not in patients with metastatic BC. These findings suggest that CTCs contribute to the release of DNA into the blood circulation. The discordance between *SOX17* methylation in CTCs and cfDNA methylation in patients with metastatic BC may be due to the release of DNA from apoptotic cells escaping from metastatic deposits at distant sites, such as bone marrow and liver [48].

Maspin, a noninhibitory member of the serine protease inhibitor superfamily, inhibits cancer cell invasion, attachment to extracellular matrices, and angiogenesis and increases sensitivity to apoptosis. However, whereas experimental data support the role of *Maspin* as a tumor suppressor, clinical data regarding its prognostic implications have led to conflicting results [49]. Sharma et al. examined its promoter methylation status in tumor tissue and matched serum samples of patients with invasive ductal BC [50]. Immunohistochemistry displayed loss of Maspin protein expression in tumor tissue that was significantly associated with methylated *Maspin* in paired serum samples. Dysregulation of DNA repair capacity is a further genetic risk factor for BC that has usually been measured in lymphocytes. In a BC case–control project, Guerrero-Preston et al., who analyzed the plasma levels of promoter methylation of *KIF1A*, *MAL*, *FKBP4*, *VGF*, and *OGDH*, found an inverse association of DNA repair capacity with promoter methylation of the kinesin motor protein KIF1A in plasma [51]. This was the first evidence of an association between genetic and epigenetic alterations in BC using blood-based tests.

In contrast to these encouraging studies, Sturgeon et al. found only insufficient differences in DNA methylation between BC cases and controls [52]. The objective of their study was to evaluate promoter methylation of a panel of 12 BC-related genes (*APC*, *BRCA1*, *CCND2*, *CDH1*, *ER1*, *GSTP1*, *HIN1*, *P16*, *RARβ*, *RASSF1*, *SFRP1*, and *TWIST*) in the serum of postmenopausal women, including BC cases with lymph node-positive disease, BC cases with lymph node-negative disease, and women with benign breast diseases. For all genes in each study group, the median levels of promoter methylation were low, typically below 5 %, but higher in lymph node-positive BC cases than in the controls. These findings demonstrate that, although there is growing evidence that methylated tumor suppressor genes in plasma/serum are promising biomarkers for BC, it is worth keeping in mind that the methylation frequency is usually lower in plasma/serum than in tumor tissues.

## Conclusions

The current studies show that quantitative and qualitative tumor-associated changes in genetic and epigenetic profiles may correlate with the biological behavior and response to therapy of BC. At present, there is a great heterogeneity amongst the cfDNA data in different studies using plasma or serum, different technical platforms and patient populations (Tables 1 and 2). Thus, a standardization of cfDNA analyses in terms of sample collection, processing, and molecular techniques is required. Then, these protocols can be validated in well-designed prospective patient cohorts providing sufficient power and sample size. Expected results could be the establishment of a multivariate risk model based on traditional clinical factors and assessment of cfDNA signatures as well as the development of companion diagnostics for targeted therapies.

Before implementation of cfDNA analysis into the clinical decision, several issues need to be addressed in future studies: (i) Because cfDNA represents mainly the genome of dying tumor cells, the time point of blood sampling during the course of a treatment is important to discover genetic alterations present in the resistant tumor cell clones. (ii) Genomic screening using NGS technologies has made enormous advances but has also led to the discovery of many genomic aberrations without known clinical relevance. Thus, clinical intervention studies with established endpoints (e.g., overall survival) in which decisions are made on the basis of cfDNA analysis are needed to demonstrate that the patient will benefit from cfDNA measurements. (iii) Masking of ctDNA by variable amounts of normal cfDNA released by dying normal cells (e.g., during chemotherapy, surgery, infections) might lead to false-negative results. (iv) Many individuals have benign tumors (e.g., skin tumors) that carry cancer-associated mutations and that may cause false-positive findings for cancer screening. Thus, the use of cfDNA analysis for primary detection of cancer requires very large cohorts of patients with cancer, and matched control individuals need to be analyzed prospectively over extended periods of time (>10 years). A possible way to circumvent this problem could be the focus on patients at high risk to develop cancer.

## Abbreviations

APC: Adenomatous polyposis coli
AUC: Area under curve
BC: Breast cancer
BEAMing: Beads, emulsion, amplification and magnetics
bp: Base pair
BRCA1: Breast cancer 1
BRF1: TFIIB-related factor
CCND2: Cyclin D2
CDKN2A (p16): Cyclin-dependent kinase inhibitor
cfDNA: Cell-free DNA
CTC: Circulating tumor cell
DUB3: Deubiquitylase
ER: Estrogen receptor
FKBP4: FK binding protein 4
GAS6: Growth arrest-specific 6
GSTP1: Glutathione S-transferase P1
HER2: Human epidermal growth factor receptor
HIN1: Hairpin induced gene 1
IDH2: Isocitrate dehydrogenase 2
JAG2: Jagged 2
KIF1A: Kinesin family member 1a
LOH: Loss of heterozygosity
MAL: Myelin and lymphocyte protein
Maspin: Mammary serine protease inhibitor
MRD: Minimal residual disease
MTA1: Metastatic tumor antigen 1
NGS: Next-generation sequencing
OGDH: Oxoglutarate dehydrogenase
PCR: Polymerase chain reaction
PIK3CA: Phosphatidylinositol-3 kinase (PI3K) catalytic subunit
PITX2: Pituitary homeobox 2
PTEN: Phosphatase and tensin homolog
RARβ: Retinoic acid receptor β
RASSF1A: Ras association domain family protein 1A
SFRP1: Secreted frizzled-related protein 1
